# Supplementary material for: Modeling Alzheimer’s disease: Bayesian copula graphical model from demographic, cognitive, and neuroimaging data
Source: J Alzheimers Dis. 2025 May 4;108(1 Suppl):S244–57. doi: 10.1177/13872877251337944 (PMC12583651; doi:10.1177/13872877251337944)
Supplement: sj-pdf-1-alz-10.1177_13872877251337944 - Supplemental material for Modeling Alzheimer’s disease: Bayesian copula graphical model from demographic, cognitive, and neuroimaging data [file sj-pdf-1-alz-10.1177_13872877251337944.pdf]

Supplemental Material

Modeling Alzheimer's disease: Bayesian copula graphical model from demographic, cognitive, and neuroimaging data

Supplemental Material 1. Variables included in the graphical model

**Category**	**Variable**	**Abbreviation**	**Type**
Volume (V)	V Hippocampus	V Hipp	Continuous[1]
	V Caudate	V Caud	Continuous[1]
	V Putamen	V Put	Continuous[1]
	V Thalamus	V Thal	Continuous[1]
	V Posterior Cingulate Cortex	V PCC	Continuous[1]
	V Precuneus	V Prec	Continuous[1]
Glucose uptake (G)	G Hippocampus	G Hipp	Continuous[1]
	G Caudate	G Caud	Continuous[1]
	G Putamen	G Put	Continuous[1]
	G Thalamus	G Thal	Continuous[1]
	G Posterior Cingulate Cortex	G PCC	Continuous[1]
	G Precuneus	G Prec	Continuous[1]
Demographic	Age	Age	Discrete
	Education	Educ	Discrete[2]
	Sex	Sex	Binary[3]
Cognitive	Memory	ADNI-MEM	Continuous
	Executive function	ADNI-EF	Continuous
Other	Number of *APOE4* alleles	*APOE4*	Binary[4]
	Amyloid score	Amy-stage	Ordinal[5]

Supplemental Table 1. All 19 variables with their corresponding name, abbreviation, and data type. [1]average of left and right brain region, [2]measured in years of formal education, [3]equal to 1 (female) or 0 (male), [4]equal to 0 (no *APOE4* alleles) or 1 (at least one *APOE4* allele), [5]ranging from 0 (little amyloid deposition) to 4 (high amyloid deposition)

Supplemental Material 2. Conditional dependencies across disease stages

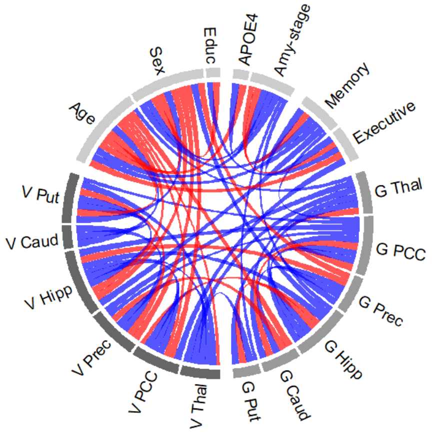

(a) All disease stages

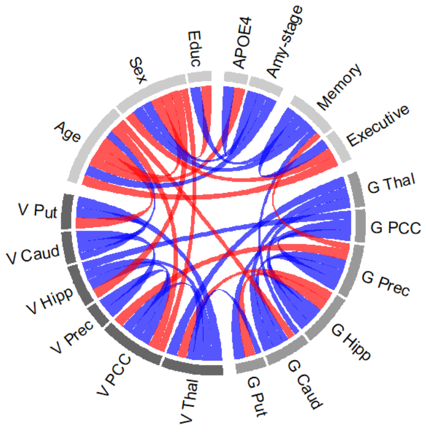

(b) Cognitively Normal

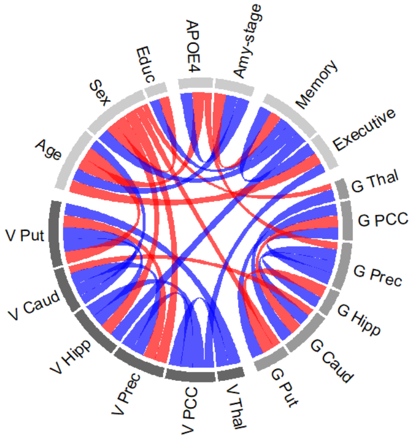

(c) Early Mild Cognitive Impairment

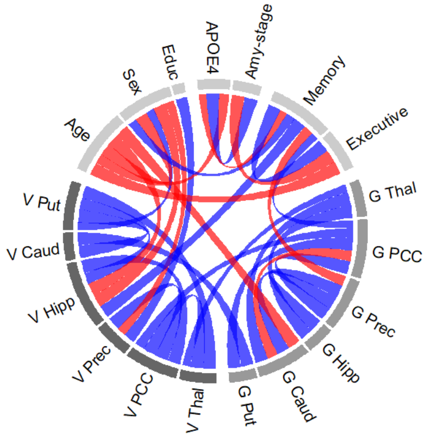

(d) Late Mild Cognitive Impairment

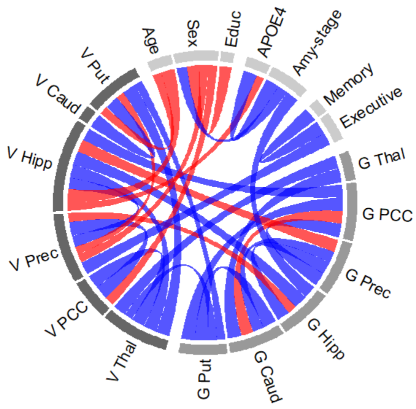

(e) AD

Supplemental Figure 1. Visualization of conditional dependence among brain-region specific glucose uptake (G), brain-region specific gray matter volume (V), and demographic variables. Figures are shown per disease stage. An edge between variables indicates a conditional dependence with a probability of at least 0.5. The width of the edges denotes the size of this probability ranging from 0.5 to 1. A blue (red) edge denotes a positive (negative) partial correlation.

Supplemental Material 3. Conditional dependencies including left and right brain regions

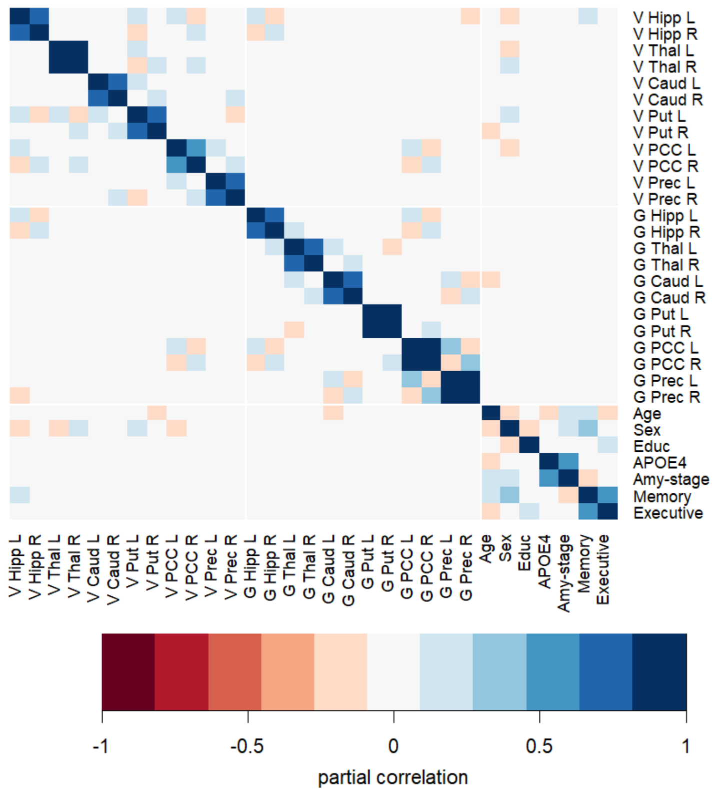

Supplemental Figure 2. Partial correlations among demographic variables, glucose uptake (G) and gray matter volume (V) of left (L) and right (R) brain regions. Partial correlations are set to zero when the corresponding edge inclusion probability is smaller than 50%.

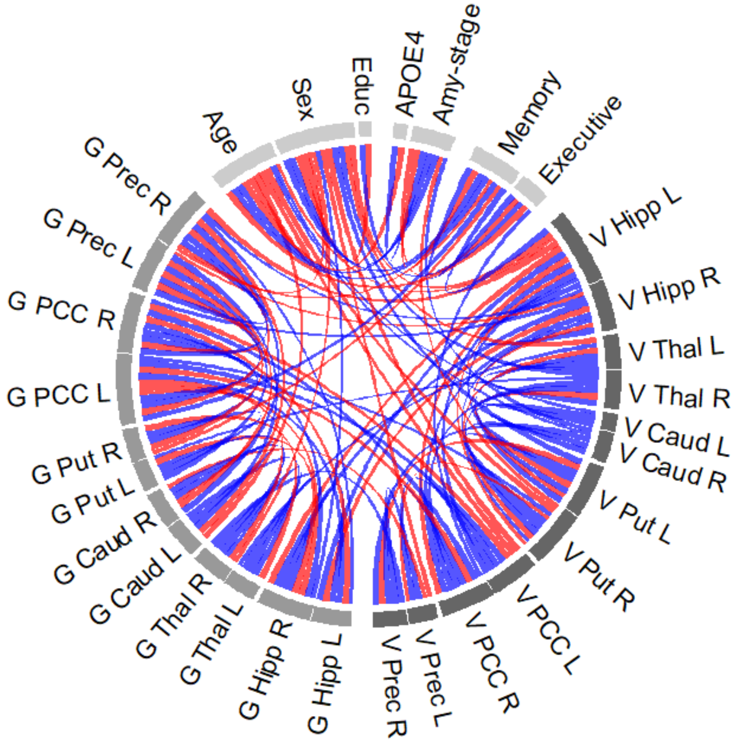

Supplemental Figure 3. Visualization of conditional dependencies between demographic variables, glucose uptake (G) and gray matter volume (V) of left (L) and right (R) brain regions. An edge between variables indicates a conditional dependence with a probability of at least 0.5. The width of the edges denotes the size of this probability ranging from 0.5 to 1. A blue (red) edge denotes a positive (negative) partial correlation

Supplemental Material 4. Conditional dependencies between brain-region specific glucose and volume

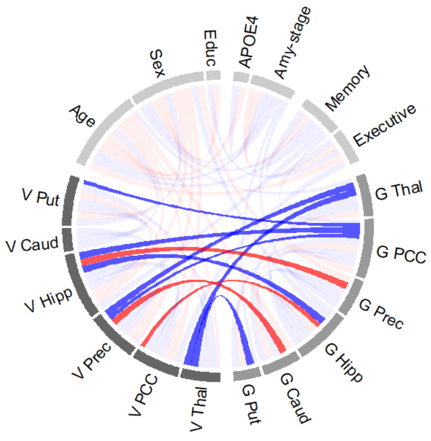

(a) All disease stages

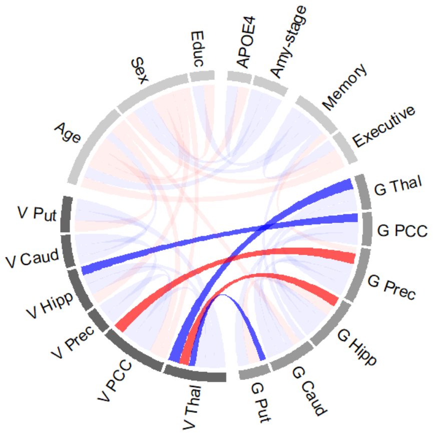

(b) Cognitively normal

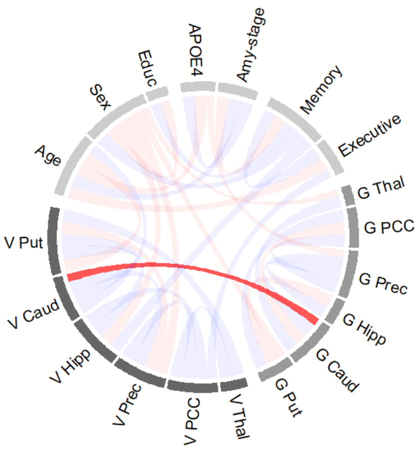

(c) Early Mild Cognitive Impairment

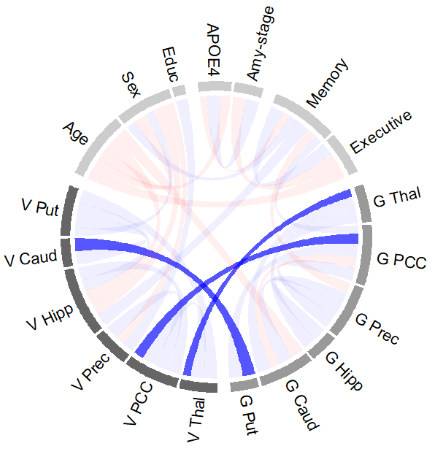

(d) Late Mild Cognitive Impairment

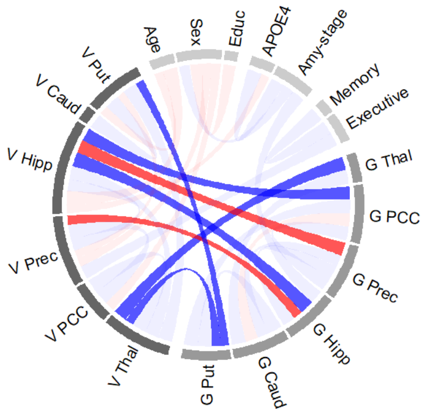

(e) AD

Supplemental Figure 4. Visualization of conditional dependencies between brain-region specific volume (V) and brain-region specific glucose uptake (G). Figures are shown per disease stage. An edge between variables indicates a conditional dependence with a probability of at least 0.5. The width of the edges denotes the size of this probability ranging from 0.5 to 1. A blue (red) edge denotes a positive (negative) partial correlation.
